# Effects of Housing Types on Cecal Microbiota of Two Different Strains of Laying Hens During the Late Production Phase

**DOI:** 10.3389/fvets.2020.00331

**Published:** 2020-06-23

**Authors:** Bishnu Adhikari, Se-Ran Jun, Young M. Kwon, Aaron S. Kiess, Pratima Adhikari

**Affiliations:** ^1^Department of Poultry Science, University of Arkansas, Fayetteville, AR, United States; ^2^Department of Biomedical Informatics, University of Arkansas for Medical Sciences, Little Rock, AR, United States; ^3^Cell and Molecular Biology Program, University of Arkansas, Fayetteville, AR, United States; ^4^Department of Poultry Science, Mississippi State University, Starkville, MS, United States

**Keywords:** hen, cecal microbiota, housing environment, egg (production), Hy-Line

## Abstract

Due to animal welfare issues, European Union has banned the use of conventional cages (CC) and non-EU countries including the US are also under constant public pressure to restrict their use in egg production. Very limited information is available on the composition of the microbial community of hens raised in different housing environments. This study was conducted to determine the effects of CC and enriched colony cages (EC) on cecal microbiota of two commercial laying hen strains, Hy-Line W36 (W36) and Hy-Line Brown (HB) during the late production stage (53, 58, 67, and 72 weeks of age). Cecal microbiota was studied by analyzing 16S rRNA gene sequences with Quantitative Insights Into Microbial Ecology (QIIME) 2 ver. 2018.8. Differentially abundant taxa were identified by Linear discriminant analysis Effect Size (LEfSe) analysis (*P* < 0.05, LDA score > 2.0). At phylum level, Actinobacteria was significantly enriched in W36 at all time points while Synergistetes (53 weeks), Spirochaetes (58 weeks), and Synergistetes and Spirochaetes (67 weeks) were significantly higher in HB. At genus level, *Bifidobacterium* (at all time points) and butyric acid producing genera such as *Butyricicoccus* and *Subdoligranulum* (58 and 72 weeks) were significantly higher in W36 as compared to HB. Moreover, Proteobacteria (72 weeks) and its associated genus *Campylobacter* (67 and 72 weeks) were significantly enriched in EC as compared to CC. Alpha diversity was significantly higher in HB (at all time points) and in EC (67 weeks) as compared to W36 and CC, respectively. Similarly, there was a significant difference in community structure (beta diversity) between W36 and HB (all time points) as well as between EC and CC (67 weeks). The effect of housing and strains was not only seen at the bacterial composition and structure but also reflected at their functional level. Notably, KEGG metabolic pathways predicted to be involved in carbohydrates degradation and amino acids biosynthesis by PICRUSt analysis were significantly different between W36 and HB housed at CC and EC. In sum, cecal microbiota composition, diversities, and their functional pathways were affected by housing type which further varied between two commercial laying hen strains, HB and W36. This suggests that both housing and genetic strains of laying hens should be considered for selection of the alternative housing systems such as enriched colony cage.

## Importance

This study addresses the microbiota profile of laying hens at their late production phase. The two most common breeds of commercial laying hens, Hy-Line W36 and Hy-Line Brown with their two different housing environments, CC and EC cage were investigated in our study. Although previous studies have investigated the performance parameters and welfare of laying hens housed in CC and EC cages, there is very limited information regarding the changes in gut microbiota, particularly in ceca. Our research findings will be important to the primary breeders to help in the decision-making process to select certain types of breed for the specific housing environment. This will further assist in exploring different feed additives and gut health enhancers that would be supplemented according to the hen performance in those two housing environments.

## Introduction

Poultry industry is the fastest-growing industry which is expected to grow continuously since the demand for meat and eggs is continuously increasing as a result of growing human population (1). In order to feed the growing human population which is expected to reach 9.8 billion by 2050 (2), there is a huge pressure to accelerate animal production including poultry. Traditionally, people focused mainly on the strategies to maximize the profit and productivity of poultry, and conventional cage (CC) system is one of those strategies developed during the 1930s and has been used in the traditional egg production since 1950s (3). Although the CC system has been considered as one of the most efficient housing methods of laying hens for a long time, it is now widely accepted that this system has negative impacts on the welfare of hens (3–7). The negative impacts of CC are mainly due to the limited space for movement that can cause musculoskeletal weakness, and low complexities of the environment, which can abolish many of their natural behaviors such as nesting, roosting, dust bathing, perching, and foraging (6–8).

Because of the increased public concerns about animal welfare, CC systems have been banned in the EU since 2012 (9). In addition, non-EU countries including USA, Canada, and Australia are also under constant public pressure to restrict the use of conventional cage systems for egg production (10). As an alternative, enriched colony cages (CC) were developed that provide more space for movement and comfort behaviors, and may allow for some dust bathing, nesting, foraging, and perching (11). Although previous studies have conducted to investigate the performance parameters and welfare of laying hens in CC and EC (5, 12), there is very limited information regarding the changes in intestinal microbiota associated with those housing systems. Furthermore, it has been shown that host factors such as breeds or strains within the same environment can affect the intestinal microbiota in chicken (13). However, those variations were less studied in laying hens in comparison to broilers. Thus, the aim of this study was to investigate the effects of CC and EC laying hen housing systems on cecal microbiota of two commercial laying hen strains, Hy-Line W-36 (W36) and Hy-Line Brown (HB).

## Materials and Methods

### Hens and Husbandry

The animal experimental protocol was approved by the Institutional Animal Care and Use Committee (IACUC) at Mississippi State University (AUP 17-554). Both strains (HB and W36) of hens were purchased from a pullet company (Mansfield Pullet Co., Missouri). Pullets ready to lay were obtained and raised in laying hen cages. Hens were reared in top two tiers of three-tiered A-frame type conventional cage (CC; dimension: 1.6′ x 2′) and both tiers of two-tired enriched colony cage (EC; dimension: 4′ x 12′; Chore-Time Inc.,) at Mississippi State University Poultry Research Farm located in Starkville, MS. Conventional cage and EC were installed in an open-sided house within the same layer house. The CC system was three-tier A-frame with a manure shield, and the EC system had two-tier with the manure belts. Both CC and EC systems consisted of galvanized wire cages with a galvanized trough-type feeder. The feeder space in CC was 15 cm/bird whereas in EC it was 22.5 cm/bird. The CC system contained two nipple drinkers per cage, and the EC system contained eight nipple drinkers per cage. The floor space in CC was 772 cm^2^ /bird whereas it was 1,505 cm^2^ /bird in the EC system. The EC system was also installed with a dark nesting area covered by non-transparent plastic curtains, perches running parallel to the cage, and a scratchpad. The scratchpad was made up of plastic. Each hen had perch space allotment of 15 cm/bird with 50 birds per cage and there were 4 perches per cage. There were 4–5 hens per nest area at one time using the nest space. Each nest area had dimension of 30 x 60 x 55 cm (lxbxh) and each EC cage had a total of two nesting areas in EC.

The design was completely randomized with 2 × 2 factorial arrangement of hen strain and cage environment. Both cages were located in the same house where A-frame cages were at the front of the house while enriched cages were at the back of the house. A-frame cages were slightly offset where the fecal material from each tier would fall directly into the pit where it was managed later to the lagoon system. Hens were housed with four hens per cage in CC and 50 per cage in EC system. There was a total of six replicates both in conventional cage and enriched colony. In conventional, we had a 6 replicate group of 12 cages with 4 hens per cage to give 288 hens of white and 288 hens of brown strain. In the enriched colony, each cage could hold 50 hens to give total 300 for each brown and white hens. Eggs were collected once a day at 1:00 p.m. daily. Hens were monitored for feed and mortality twice a day but eggs were collected from both cage systems once a day. The lighting schedule was 16 h light and 8-h darkness and commercial laying hen ration were provided *ad libitum* according to the Hy-Line management guide recommendation containing 2,760 Kcal ME/kg and 16% CP (Table 1).

**Table 1:** Diet formulation and calculated composition of diet fed to Hy-Line hens.

**Ingredient**	**Amount (%)**
Corn	57.00
Soybean meal	21.79
Limestone	11.06
Corn DDGS	5.00
Poultry fat	2.84
Dicalcium phosphate	1.44
Common salt	0.30
DL-Methionine	0.23
Vitamin premix	0.13
Mineral premix	0.13
L-Lysine HCL	0.09
Total	100.00
**Calculated composition**	
ME (Kcal/kg)	2.760
CP (%)	16
Ca (%)	4.6
Available *P* (%)	0.40

### Cecal Microbiota Analysis

#### Sample Collection and Processing

At 53, 58, 67, and 72 weeks of age, six hens per group were humanely euthanized with CO_2._ One cecum from each hen was collected aseptically and stored at −20°C until microbiota analysis. The number of samples from each group used for microbiota analysis is summarized in Table 2.

**Table 2:** Summary of samples and reads distribution across different groups.

**Variables**	**53 weeks**	**58 weeks**	**67 weeks**	**72 weeks**
**HOUSE**				
CC	63,697.3 ± 4,212.0 (12)	61,876.4 ± 5,055.5 (9)	56,769.6 ± 3,649.8 (12)	59,954.4 ± 3,174.7 (12)
EC	66,289.4 ± 3707.3 (12)	61,392.5 ± 2,759.5 (11)	58,962.2 ± 2,287.3 (12)	66,829.9 ± 3,865.4 (10)
**STRAIN**				
HB	67,706.5 ± 3,773.8 (12)	56,247.0 ± 3,870.6 (11)	56,402.4 ± 2,576.2 (12)	59,455.7 ± 3,996.4 (11)
W-36	62,280.2 ± 4,024.3 (12)	68,165.3 ± 2,134.0 (9)	59,329.3 ± 3427.1 (12)	66,703.5 ± 2,840.0 (11)
**HOUSE-STRAIN**				
CC-HB	62,531.5 ± 6,031.7 (6)	54,635.2 ± 7409.1 (5)	54,942.5 ± 3,271.5 (6)	58,006.8 ± 5,730.9 (6)
CC-W36	64,863.0 ± 6,413.6 (6)	70,928.0 ± 3,563.5 (4)	58,596.7 ± 6,824.6 (6)	61,902.0 ± 3,160.0 (6)
EC-HB	72,881.5 ± 3,954.2 (6)	57,590.2 ± 4,188.2 (6)	57,862.3 ± 4,200.8 (6)	61,194.4 ± 6,098.6 (5)
EC-W36	59,697.3 ± 5,239.9 (6)	65,955.2 ± 2,455.0 (5)	60,062.0 ± 2,211.1 (6)	72,465.4 ± 3,763.7 (5)

*The number in brackets represent number of samples used for microbiota analysis. The values in each cell represent an average number of reads/sample (Mean±SE) that group*.

#### DNA Extraction, PCR, and Library Preparation for Sequencing

Quick-DNA™ Fecal/Soil Microbe Kits (Catlog No. D6012, ZymoResearch, USA) was used to extract genomic DNA from ~150 mg of cecal content per sample following the manufacturer's instructions. V4 region of 16S rRNA gene from genomic DNA of each sample was amplified using the primers 515F (14) and 806R (15). The library of amplicons for sequencing was prepared according to the 16S Illumina PCR protocol described in the Earth Microbiome project (http://www.earthmicrobiome.org) (16) with slight modifications. In brief, Platinum™ II Hot-Start Green PCR Master Mix (2X) (Thermofisher Scientific, Catalog No. 14000013) was used to conduct PCR in a 25 μl final reaction volume through 30 cycles. The thermocycling condition of PCR consisted of an initial denaturation step at 94°C for 2 min, 35 cycles of 0.5 min at 94°C, 0.5 min at 60°C, and 0.5 min at 68°C, and a final extension of 5 min at 68°C.

The length of amplified products was confirmed with 1% agarose gel electrophoresis and equal amounts (~300 ng) of amplicons from all sample as measured by Qubit dsDNA BR Assay Kit (ThermoFisher Scientific, Catalog No. Q32850) were pooled together. The pooled amplicons were finally run on 1% agarose gel electrophoresis, purified using Zymoclean Gel DNA Recovery Kit (Zymo Research, Catalog No. D4007), and sequenced with Illumina MiSeq paired end 300 cycle options at University of California at Davis.

### Data Analysis

#### Egg Data Analysis

One way ANOVA was used to analyze egg production data using SAS 9.2 version. Mean separation was performed using Fisher's Protected LSD. A statistical *P*-value of either less or equal to 0.05 was considered significant.

#### Amplicons Sequence Analysis

Nebula cloud computing platform of the University of Arkansas was used to process raw sequencing reads in QIIME 2 version 2018.8 (17) utilizing the pipelines developed for paired-end data types. In sum, “demux emp-paired” method of q2-demux plugin was used to demultiplex sequencing reads followed by quality filtering and denoising with “dada2 denoise-paired” method of q2-dada2 (18) plugin available at QIIME 2. The truncation length of forward and reverse reads was set at 240 and 200 bp, respectively, which is based on the quality score criteria (≥30). Taxonomic assignments was performed using a Naive Bayes classifier (19) pre-trained with Greengenes (Version 13.8) 99% OTUs (20) and q2-feature-classifier plugin, where the sequences have been trimmed to include only the V4 region of the 16S rRNA gene bound by the 515F/806R primer pair. The core-metrics-phylogenetic method at a sampling depth of 31,060 was used to analyze Alpha and Beta diversity. Shannon's diversity index (21) and UnWeighted UniFrac distance metric (22) were used to calculate alpha and beta diversity, respectively. All figures including Principle Coordinate Analysis (PCoA) plot were created from ggplot2 package of R (23).

Statistical differences among treatment groups at different taxonomic assignments were calculated using LEfSe (24) using criteria*, P* < 0.05, LDA score > 2.0. While comparing taxa between two hen strains, hen strains were used as Class and Housing types were used as subclass. Likewise, Housing types were used as Class and hen strains as subclass while comparing taxa between housing types. The significant differences in alpha diversity were calculated using the alpha-group-significance command of QIIME2 which uses Kruskal-Wallis test. On the contrary, statistical differences in beta diversity among groups were calculated by PERMANOVA test (25) using the beta-group-significance command of QIIME2 with pairwise comparison option. For both diversity analyses, the corrected *P*-values for multiple comparisons (*q*) were used to report a significant difference between two groups, where the level of significance was set at *q* < 0.05. PICRUSt2 (26) was used to predict the metabolic pathways of cecal microbiota, and MetaCyc database (27) was used to describe the predicted pathways. Differentially abundant features were identified using Welch's *t*-test built-in STAMP software (28), where features were filtered using *P* < 0.05 and difference in mean proportions (%) <0.03 criteria.

## Results

### Cecal Microbiota and Egg Production

Summarization of the feature table resulted in 5,568,578 sequence reads from 90 samples that ranged from 31,060 to 88,097 reads per sample. The median and mean±SE reads per sample were 63,893.50 and 61,873.09 ± 1,270.94, respectively. In addition, there were altogether 1,759 unique features (amplicon sequence variants) from these 90 samples. The summary of average reads per sample in different groups is summarized in Table 2. There was a significant interaction effect of house and strain type at 53 weeks, where W36 had significantly higher hen-day egg production (HDEP) as compared to the HB raised in CC housing (89 vs. 72%, *P* < 0.05). At 58 and 67 weeks, although the HDEP of W36 was numerically higher than HB, no significant differences were observed. At 72 weeks, the main effect of strain was observed, where HDEP of W36 was significantly higher as compared to HB (65 vs. 56%). The production in both cage types were within the range of the Hy-Line Management guide (Hy-Line International).

### Cecal Microbiota Composition at the Phylum Level

**Figure 1:**
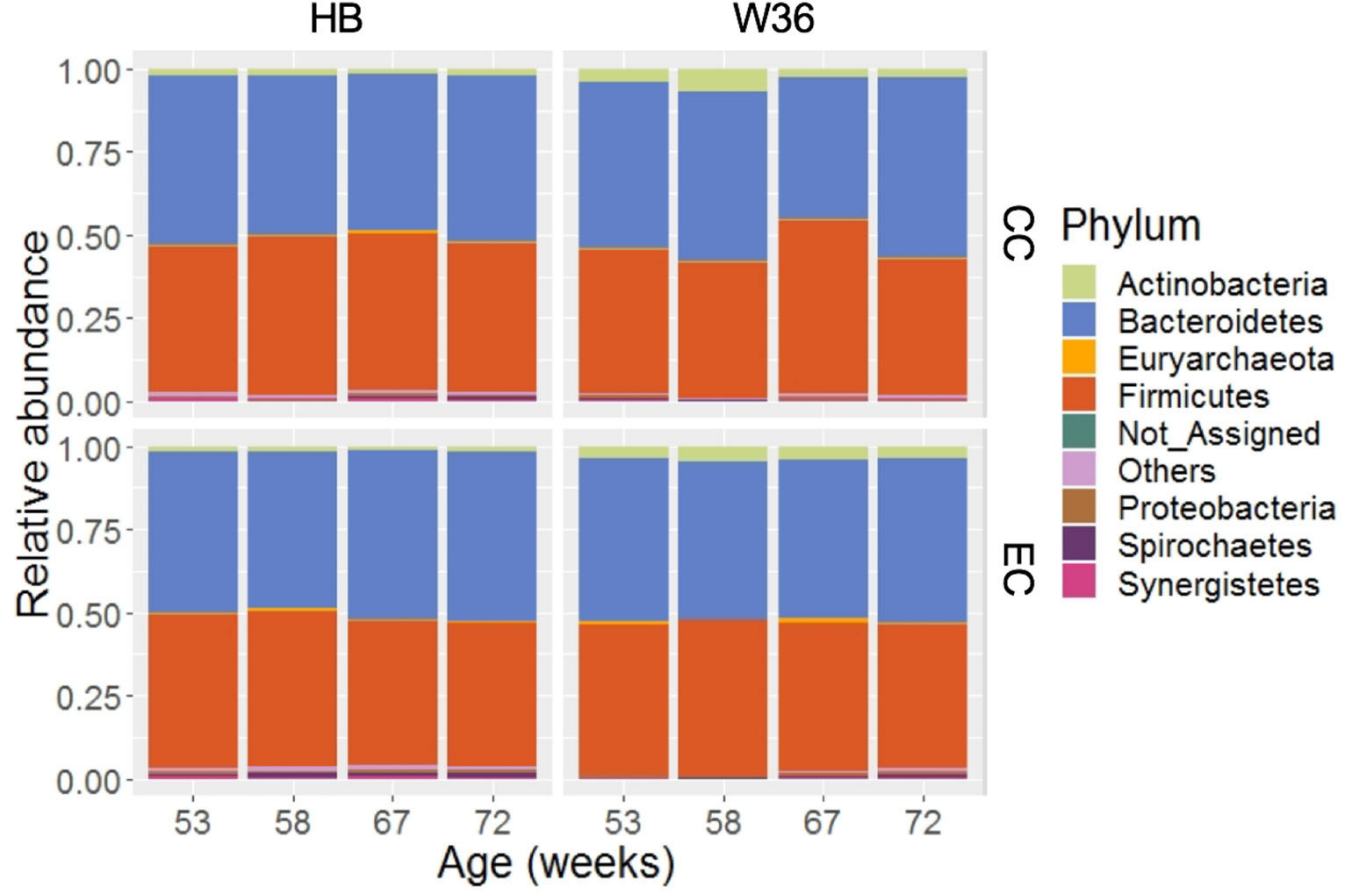
The relative abundance of cecal microbiota at phylum level. HB and W36 represent Hy-Line Brown and Hy-Line W-36, while CC and EC represent Conventional Cage and Enriched Colony Cage, respectively. Not_Assigned represent the reads that weren't assigned at any phyla, where “Others” represent the phyla which were present less than <0.4% on average of all samples.

Taking consideration of all samples, 99.36% of total sequence reads were assigned to 15 different bacterial phyla, while 0.63% of total sequence reads were assigned to domain Archaea. In addition, 0.01% of total sequence reads were assigned to Kingdom Bacteria but not to the lower level of the taxonomy. Among those phyla, Bacteroidetes (49.05%) was the predominant phylum followed by Firmicutes (45.05%). Other important phyla with relative abundance greater than 0.2% included Actinobacteria (2.70%), Proteobacteria (0.77%), Spirochaetes (0.52%), Synergistetes (0.41%), and WPS-2 (0.34%). The relative abundance levels of major phyla that were presented in two different housing types at four different time points are shown in Figure 1. The relative abundance of Bacteroidetes was the highest followed by Firmicutes in both HB and W36 irrespective of housing types and ages, except in W36 hens housed in CC housing at 67 weeks where the Firmicutes (51.96%) was found as the predominant phylum (Figure 1). Likewise, the relative abundance of Actinobacteria was found higher especially in W36 irrespective of housing as shown in Figure 1.

### Differentially Abundant Phyla

The differentially abundant phyla in two different hen strains and housing types as identified by LEfSe (*P* < 0.05 and LDA score > 2.0) are summarized in Table 3. The phylum Actinobacteria was significantly enriched in W36 group throughout all four different ages as compared to the HB group. However, the phyla Synergistetes and Spirochaetes were significantly abundant in HB group at 53 and 58 weeks, respectively, and both Synergistetes and Spirochaetes at 67 weeks as compared to W36. At 72 weeks, no significant difference was observed at any phyla between HB and W36 groups. Regarding housing effects, the phylum Spirochaetes was significantly higher in EC group in both 53 and 58 weeks as compared to CC. On the contrary, Bacteroidetes and Firmicutes were significantly enriched in EC and CC group, respectively, at 67 weeks. At 72 weeks, Proteobacteria was significantly higher in EC as compared to CC group.

**Table 3:** Summary of differentially abundant phyla identified by LEfSe (*P* < 0.05, LDA score > 2.0).

**Group**	**53 weeks**	**58 weeks**	**67 weeks**	**72 weeks**
**HOUSE**				
CC	–	–	Firmicutes	–
EC	Spirochaetes	Spirochaetes	Bacteroidetes	Proteobacteria
**STRAIN**				
HB	Synergistetes	Spirochaetes	Synergistetes, Spirochaetes	–
W36	Actionobacteria	Actionobacteria	Actionobacteria	Actionobacteria

### Cecal Microbiota Composition at the Genus Level

**Figure 2:**
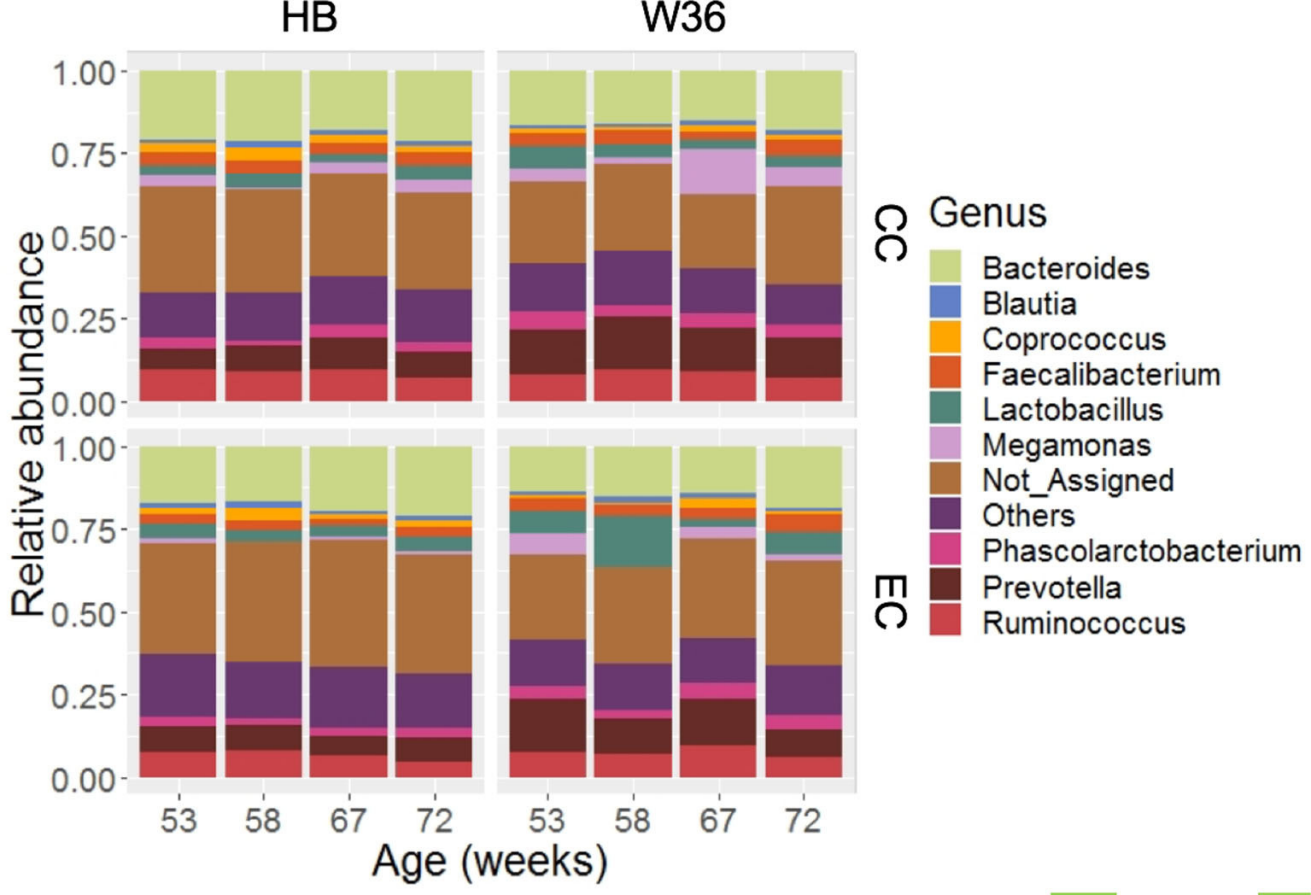
The relative abundance of cecal microbiota at genus level. HB and W36 represent Hy-Line Brown and Hy-Line W-36, while CC and EC represent Conventional Cage and Enriched Colony Cage, respectively. Not_Assigned represent the reads that weren't assigned at genus but assigned at higher taxonomic level. Others represent the genera which were present less than <1.0% on average of all samples.

Out of 99.36% of total sequence reads that were assigned to one of the bacterial phyla, 68.45% were properly assigned to one of the 89 bacterial genera while taking account of all samples. The remaining reads were assigned to higher level of bacterial taxa such as family, order, class, and phylum. Among those genera, *Bacteroides* (17.60%) was the predominant genus, followed by *Prevotella* (10.20%), *Ruminococcus* (7.91%), *Lactobacillus* (4.83%), *Fecalibacterium* (3.60%), *Phascolarctobacterium* (3.41%) and *Megamonas* (3.37%). Other notable genera included *Coprococcus, Blautia, Peptococcus*, genus S24-7, and *Turicibacter* whose relative abundance ranged from 1.21 to 1.91%. The relative abundance of major genera that were presented in two different housing types and strains at four different time points are shown in Figure 2. *Bacteroides* that ranged from 13.57% (EC-W36 at 53 weeks) to 21.69% (CC-HB at 58 weeks) was the predominant genus in both hen strains housed in either CC or EC except in W36 housed at EC at 53 and 58 weeks, where *Prevotella* (16.13%) and *Lactobacillus* (15.65%) were the predominant genera in respective ages (Figure 2). The relative abundance of *Prevotella* ranged from 5.49 to 9.78% in HB (Figure 2; left half), whereas it ranged from 8.62 to 16.13% in W36 (Figure 2; right half). Similarly, the relative abundance of *Ruminococcus* ranged from 4.83 to 9.75% in HB, while it ranged from 5.93 to 9.84% in W36. In addition, *Lactobacillus* ranged from 2.59 to 4.72% in HB, but it ranged from 2.35 to 15.65% in W36. Another important observation was the genus *Megamonas* which was found the highest (13.75%) in W36 housed at 67 weeks in CC housing.

### Differentially Abundant Genera in Two Different Hen Strains

**Figure 3:**
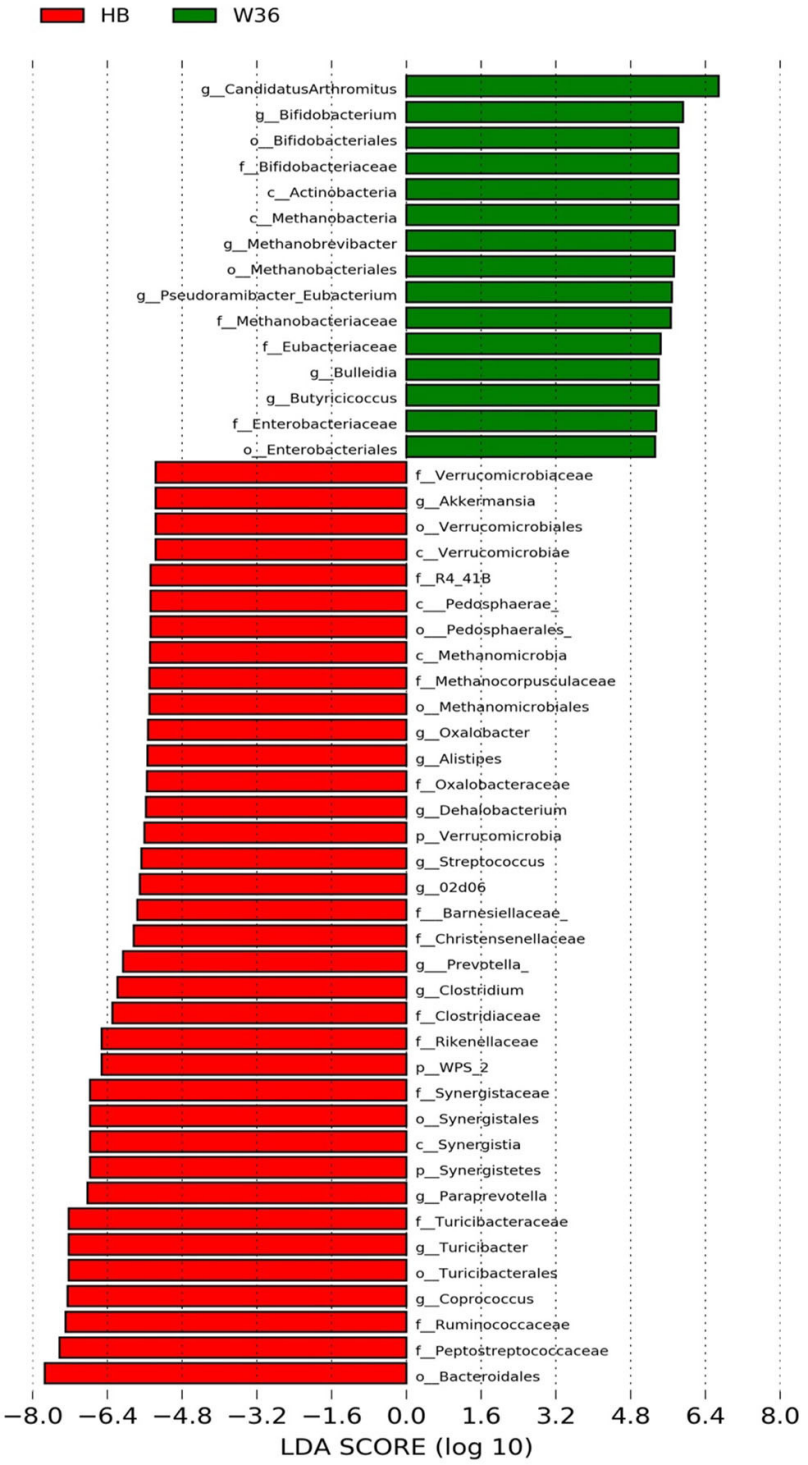
Differentially abundant taxa that were assigned at the genus level and identified by LEfSe (*P* < 0.05, LDA score > 2.0) between Hy-Line Brown (HB) and Hy-Line W-36 (W36) at 53 weeks.

**Figure 4:**
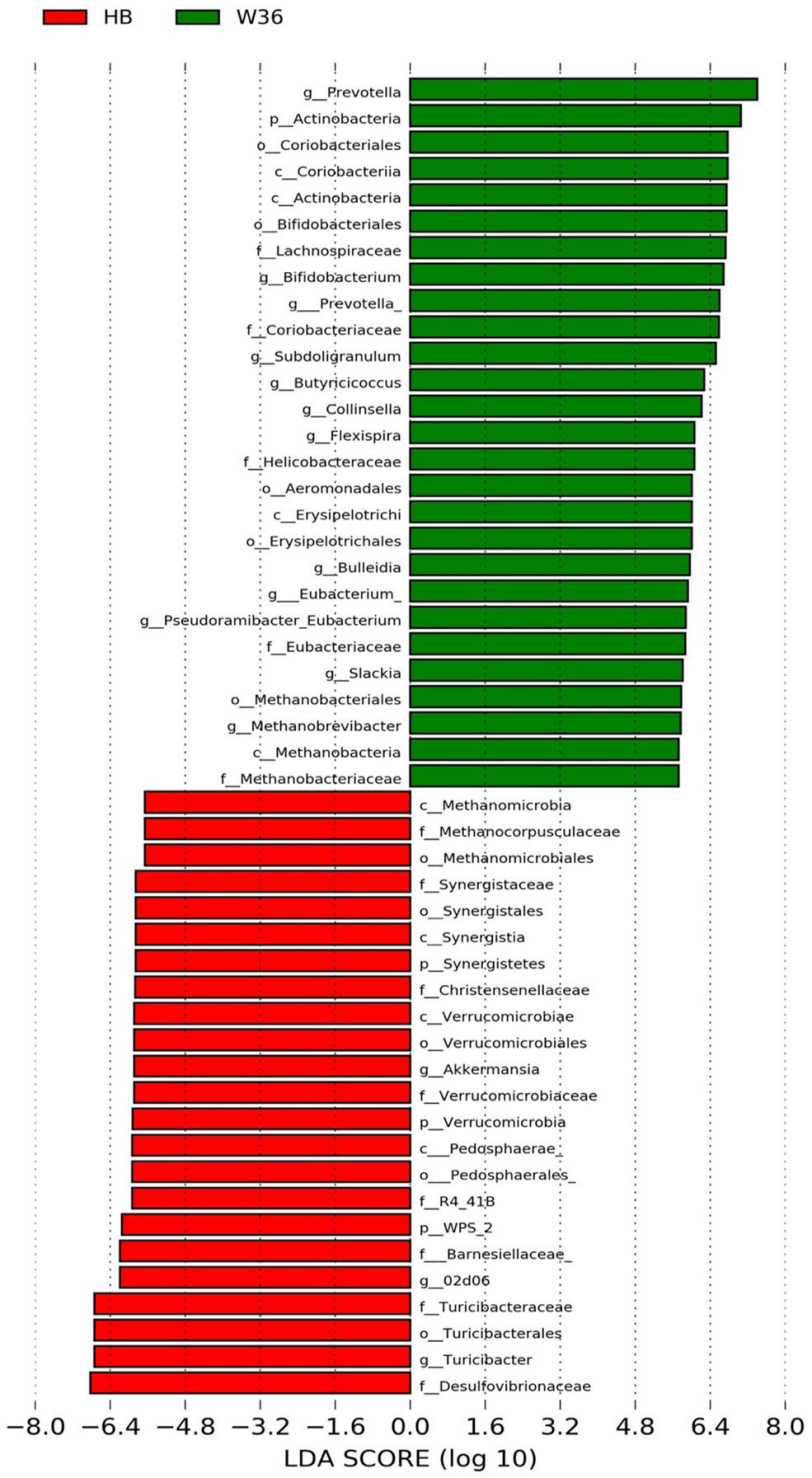
Differentially abundant taxa that were assigned at the genus level and identified by LEfSe (*P* < 0.05, LDA score > 2.0) between Hy-Line Brown (HB) and Hy-Line W-36 (W36) at 58 weeks.

**Figure 5:**
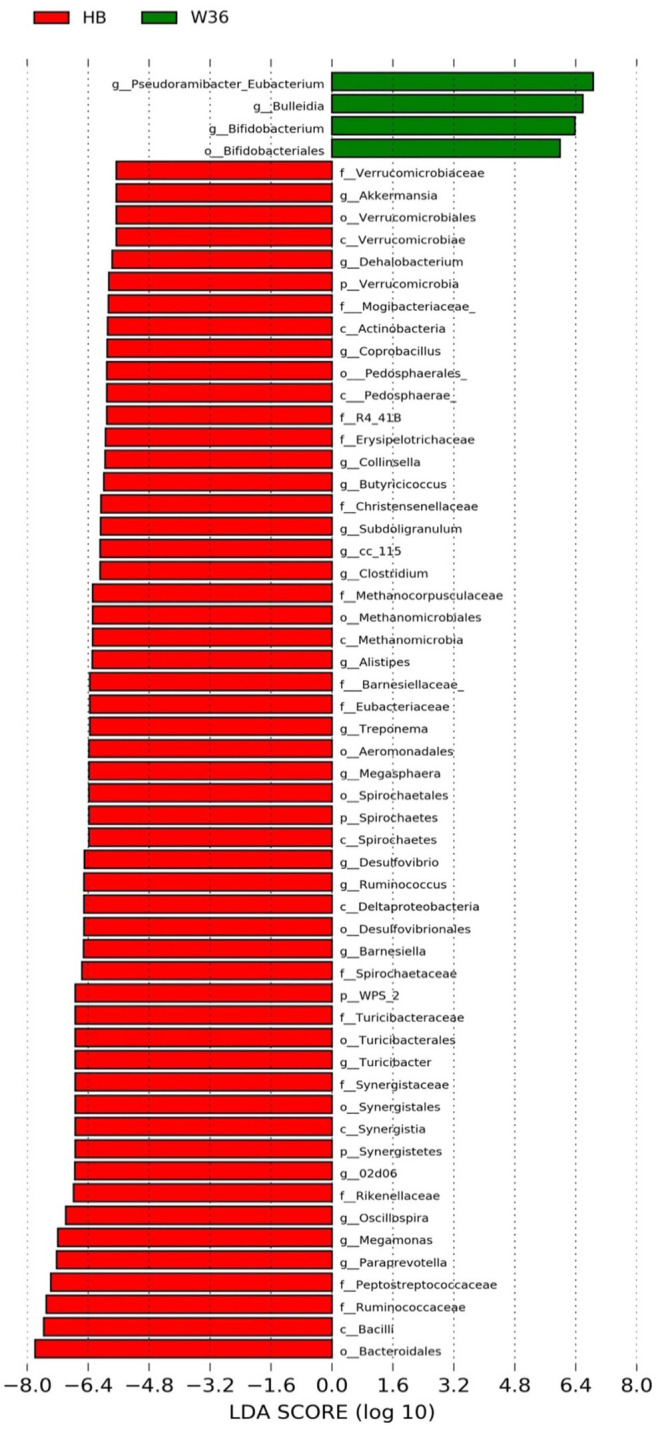
Differentially abundant taxa that were assigned at the genus level and identified by LEfSe (*P* < 0.05, LDA score > 2.0) between Hy-Line Brown (HB) and Hy-Line W-36 (W36) at 67 weeks.

**Figure 6:**
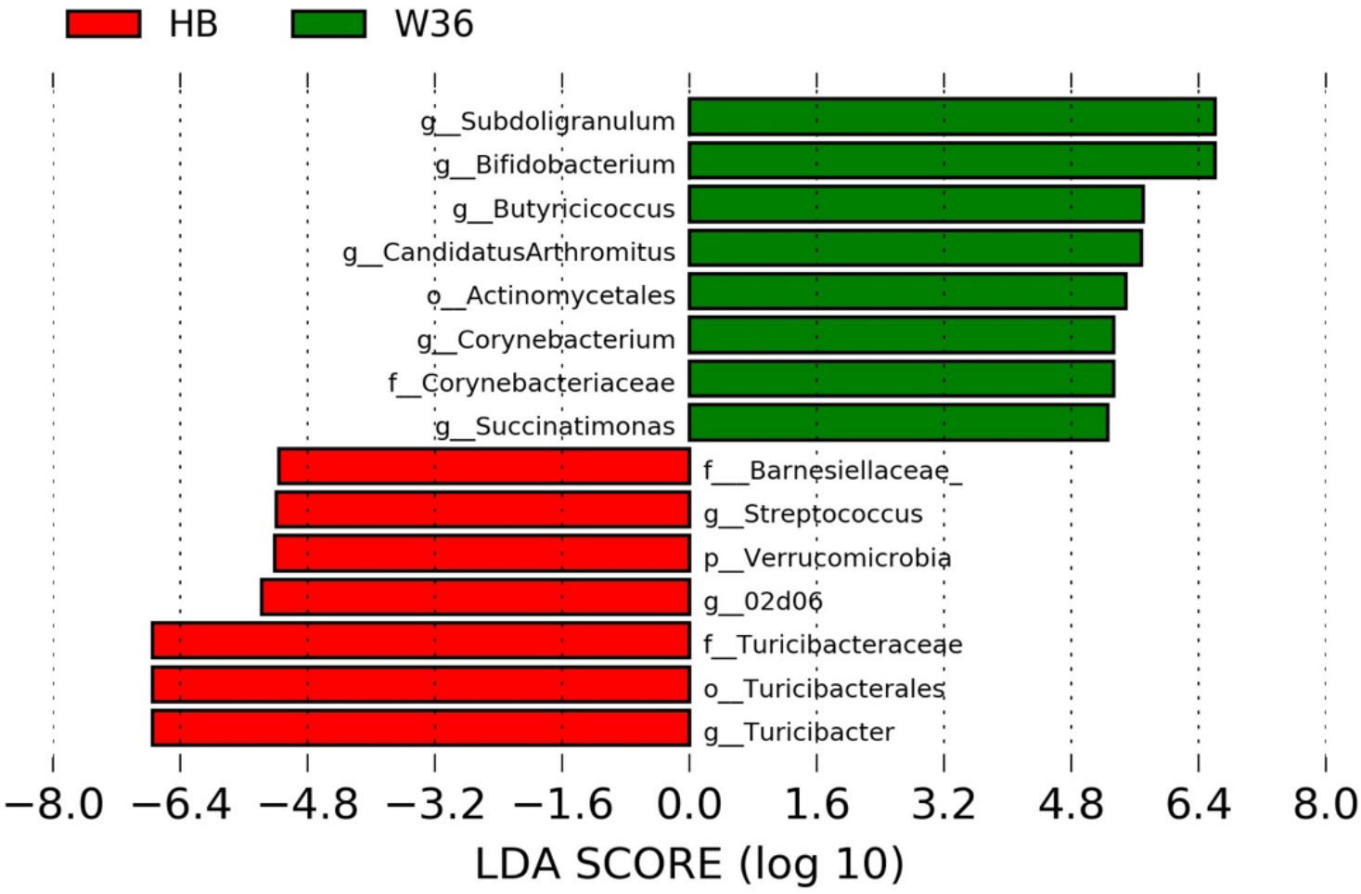
Differentially abundant taxa that were assigned at the genus level and identified by LEfSe (*P* < 0.05, LDA score>2.0) between Hy-Line Brown (HB) and Hy-Line W-36 (W36) at 72 weeks.

The strain effect was more pronounced than housing effect, and the bacterial taxa that were differentially abundant between W36 and HB strains at 53, 58, 67, and 72 weeks are shown in Figures 3–6, respectively. The number of bacterial taxa at the genus level that was significantly higher in W36 was 15, 27, 4, and 8 at 53, 58, 67, and 72 weeks, respectively. The genus *Bifidobacterium* was significantly enriched in W36 as compared to HB throughout all time points. In addition, *Butyricicoccus* (except, 67 weeks), unidentified genera of phylum Actinobacteria (except, 67 weeks), *Bulleidia* and *Pseudoramibacter-Eubacterium* (except 72 weeks) were significantly higher in W36 at all time points. Other notable genera that were significantly abundant in W36 were *Candidatus Arthromitus* (except 58 and 67 weeks) and *Subdoligranulum* (except 53 and 67 weeks) as shown in Figures 3–6. Moreover, *Prevotella, Collinsella, Flexispira*, and *Slackia* were presented significantly higher in W36 only at 58 weeks (Figure 4), whereas *Succinatimonas* was presented significantly higher only at 72 weeks (Figure 6).

On the contrary, the numbers of bacterial taxa at the genus level that was significantly higher in HB were 36, 21, 54, and 7 at 53, 58, 67, and 72 weeks, respectively. *Turicibacter*, genus 02d06 of Clostridiaceae family, the unidentified genus that belongs to family Barnesiellaceae, and that belong to phylum Verrucomicrobia were significantly enriched in HB throughout all time points as shown in Figures 3–6. In addition, the genus *Akkermansia*, and the unidentified genera that belong to phylum Synergistetes, and that belong to family Christensenellaceae were also significantly higher in HB at all time points except at 72 weeks. Similarly, *Paraprevotella, Clostridium, Dehalobacterium*, and the unidentified genera that belong to family Ruminococcaceae, Preptostreptococcaceae, and that belong to order Bacteroidales were significantly higher in HB as compared to W36 at both 53 and 72 weeks. Moreover, *Megamonas, Oscillospira, Desulfovirbrio, Megasphaera, Treponema, Alistipes*, cc_115, *Butryricicoccus, Collinsella*, and *Coprobacillus* were presented significantly higher in HB, but only at 67 weeks of age.

Interestingly, some of the archaeal taxa were also found to be differentially presented between two strains of laying hens throughout all time points except at 72 weeks. Methanobrevibacter and 3 unknown genera that were assigned as Methanobacteria, Methanobacteriales, and Methanobacteriaceae, respectively, were significantly higher in W36 at 53 and 58 weeks, while unknown genera that were assigned as Methanomicrobia, Methanomicrobiales, and Methanocorpusculaceae were significantly higher in HB (except 72 weeks).

### Differentially Abundant Genera in Two Different Housing Types

**Figure 7:**
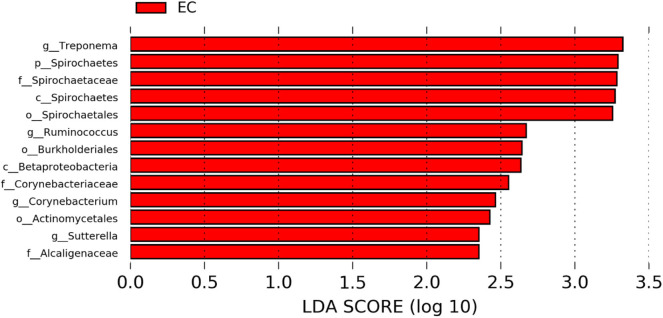
Differentially abundant taxa that were assigned at the genus level and identified by LEfSe (*P* < 0.05, LDA score > 2.0) between Conventional Cage (CC) and Enriched Colony Cage (EC) housing systems at 53 weeks.

**Figure 8:**
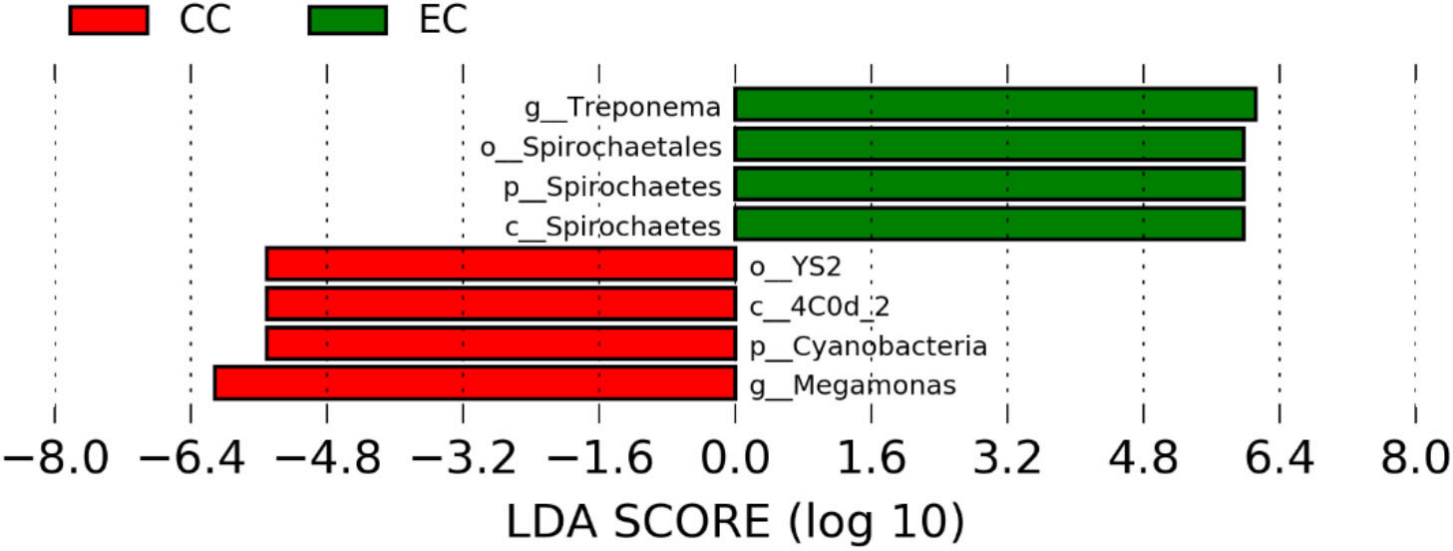
Differentially abundant taxa that were assigned at the genus level and identified by LEfSe (*P* < 0.05, LDA score>2.0) between Conventional Cage (CC) and Enriched Colony Cage (EC) housing systems at 58 weeks.

**Figure 9:**
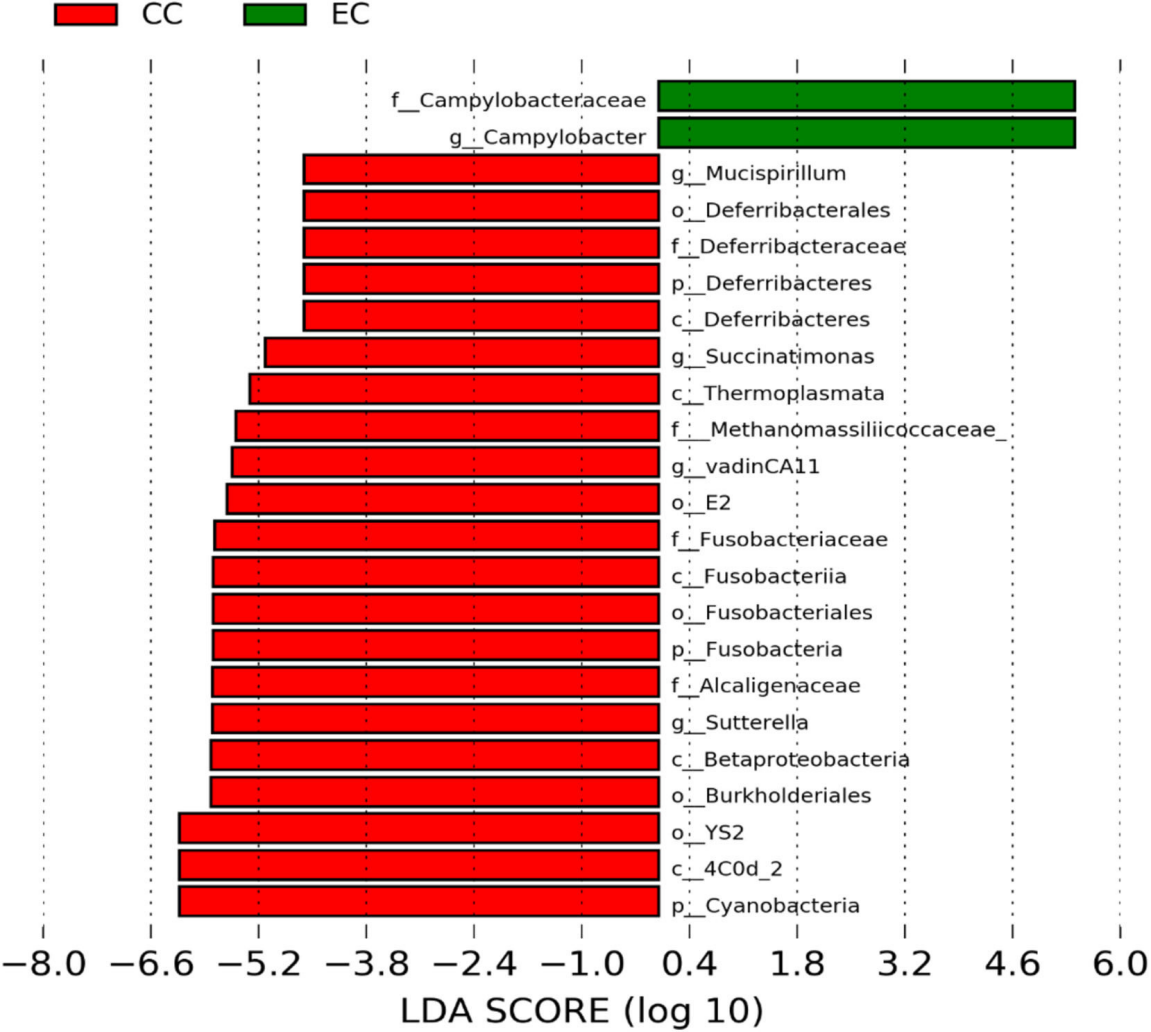
Differentially abundant taxa that were assigned at the genus level and identified by LEfSe (*P* < 0.05, LDA score > 2.0) between Conventional Cage (CC) and Enriched Colony Cage (EC) housing systems at 67 weeks.

**Figure 10:**
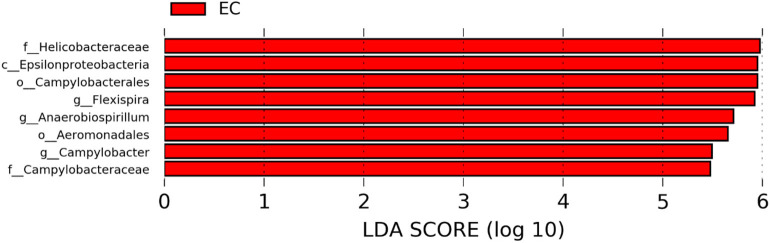
Differentially abundant taxa that were assigned at the genus level and identified by LEfSe (*P* < 0.05, LDA score > 2.0) between Conventional Cage (CC) and Enriched Colony Cage (EC) housing systems at 72 weeks.

The significantly abundant bacterial taxa at genus level which are identified by LEfSe between two housing types at 53, 58, 67, and 72 weeks are shown in Figures 7–10, respectively. At 53 and 72 weeks of age, the significantly abundant bacterial taxa were found only with EC housing, while at 58 and 67 weeks, both housing types showed differentially abundant bacterial taxa. The bacterial genus *Treponema* and the unknown genera of order Spirochaetales, Spirochaetes, and Spirochaetes were significantly enriched in EC as compared to CC at both 53 and 58 weeks. On the contrary, *Campylobacter* and other unknown genera of family Campylobacteraceae were significantly higher in EC at both 67 and 72 weeks. In addition, bacterial genera such as *Ruminococcus, Corynebacterium, Sutterella*, and the unknown genera that were assigned at order Burkholderiales and Actinomycetales, and family Corynebacteriaceae and Alcaligenaceae were significantly abundant in EC at 53 weeks. Similarly, the genus *Flexispira, Anaerobiospirillum*, and unknown genera that were assigned at family Helicobacteraceae were significantly enriched in EC at 72 weeks.

However, the differentially enriched bacterial taxa in CC were observed only at 58 and 67 weeks with more number at 67 weeks. At both 58 and 67 weeks, the unknown genera that were assigned at class 4c0d_2 and order YS2 of phylum Cyanobacteria were significantly higher in CC as compared to the EC. In addition, Megamonas was significantly higher in CC at 58 weeks, while genera such as Mucispirillum, Succinatimonas, and Sutterella were significantly higher at 67 weeks.

### Alpha Diversity

**Figure 11:**
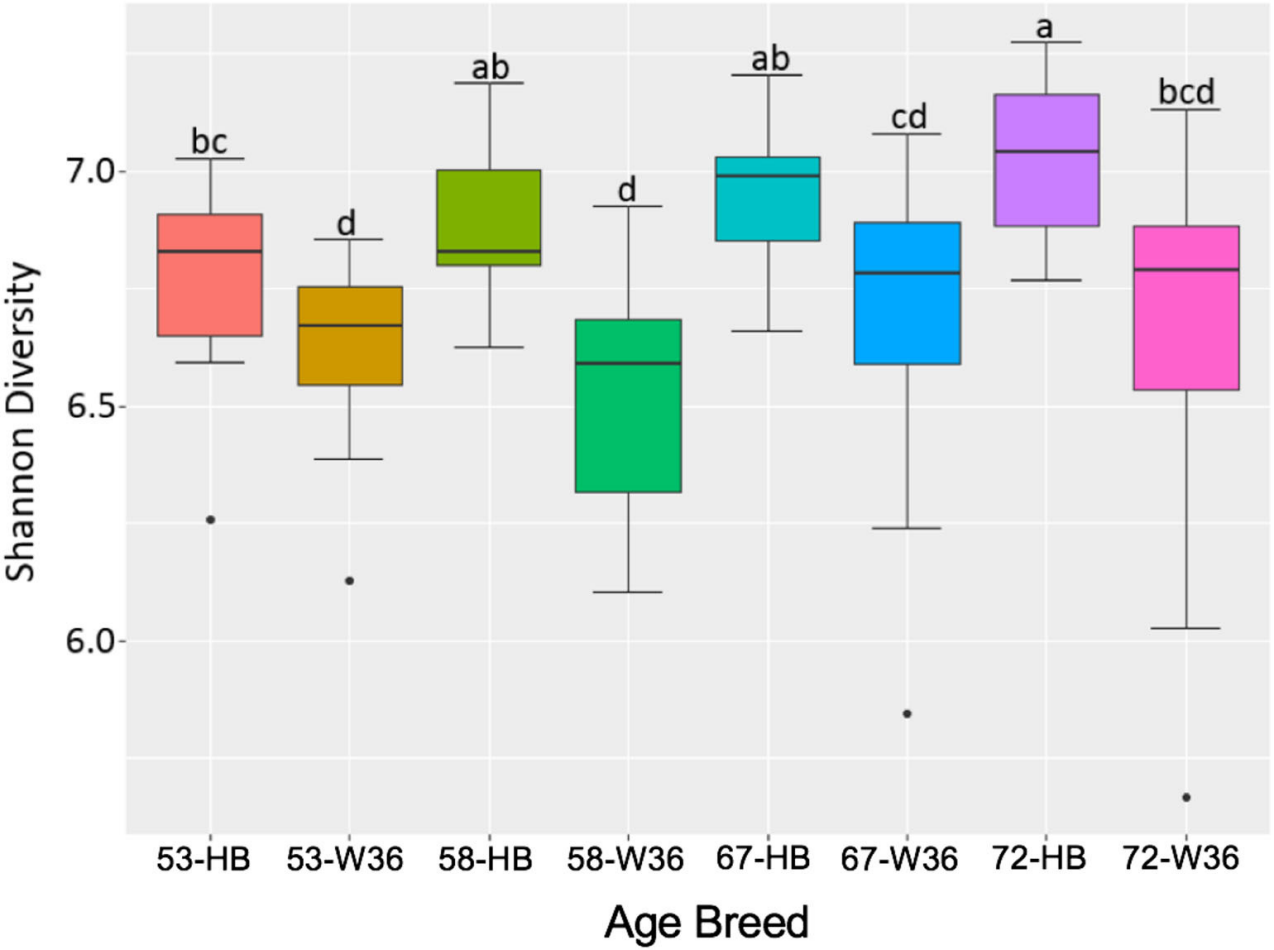
The difference in alpha diversity as measured by Shannon's diversity between Hy-Line Brown (HB) and Hy-Line W-36 (W36) at 53, 58, 67, and 72 weeks of hens' ages.

**Figure 12:**
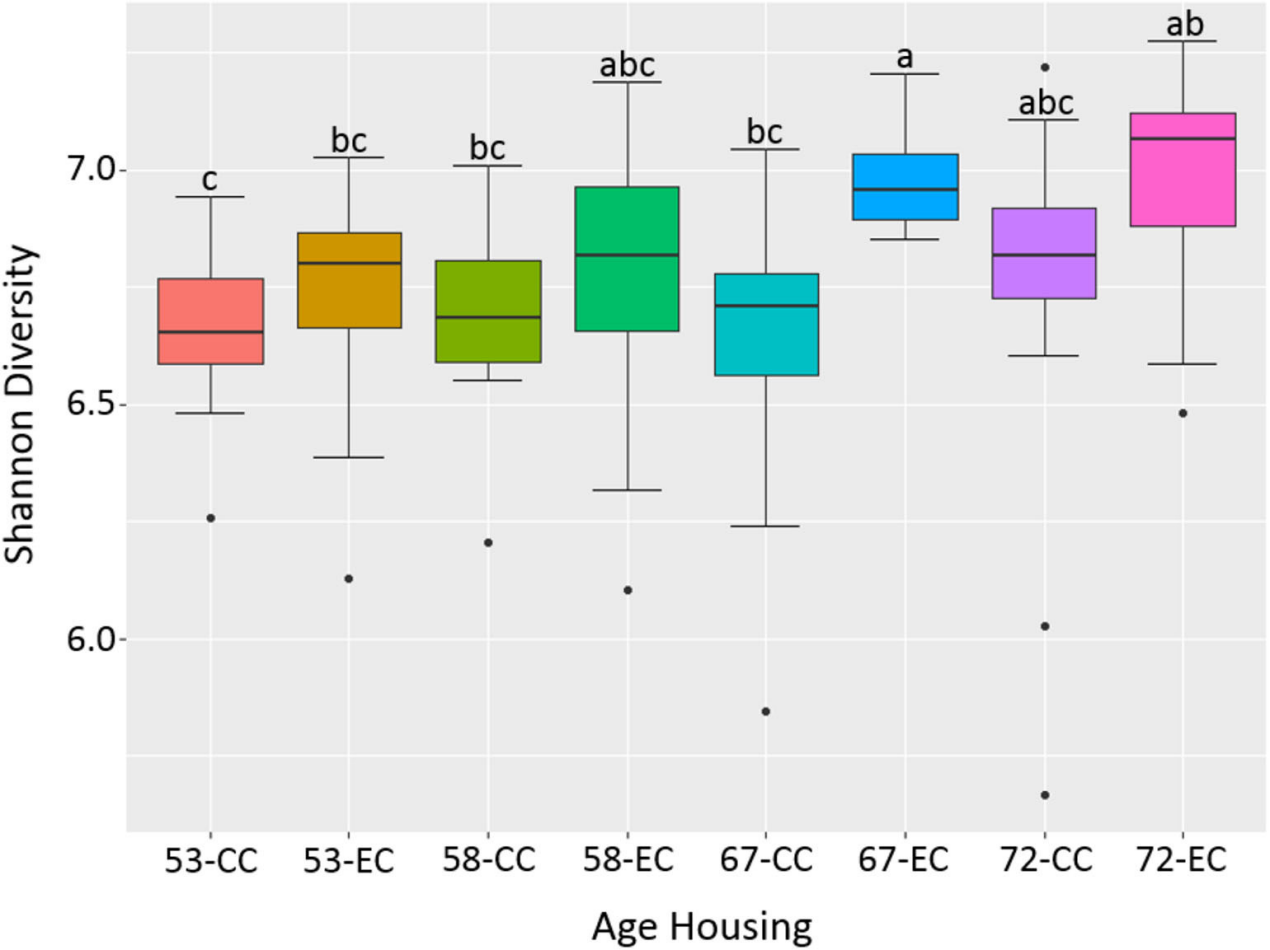
The difference in alpha diversity as measured by Shannon's diversity between hens housed in Conventional Cage (CC) and Enriched Colony Cage (EC) systems at 53, 58, 67, and 72 weeks of hens' ages.

The bacterial diversity within a group (alpha diversity) was calculated by Shannon index. The significant differences were determined between the two groups at the adjusted *P-*value (*q*) < 0.05. The alpha diversities for two different strains and housing types across four different ages of birds are shown in Figures 11 and 12, respectively. The alpha diversity was highly affected by strains in comparison to housing. The alpha diversities in HB strain was significantly higher as compared to W36 throughout all four ages as shown in **Figure 14**. The alpha diversity increased with increase in age of both strains which was more noticeable in HB, where the alpha diversity of HB strain at 72 weeks was significantly higher in comparison to HB at 53 weeks of age as shown in Figure 11.

Similarly, as age of birds increased, the alpha diversity also increased in both housing types with more pronounced increase in EC housing, where the alpha diversity of birds at 67 weeks was significantly higher as compared to those at 53 weeks as shown in Figure 12. Although the alpha diversities in birds housed in EC were numerically higher in comparison to those housed in CC across all four ages, the significant difference between EC and CC was found only at 67 weeks of age.

### Beta Diversity

**Figure 13:**
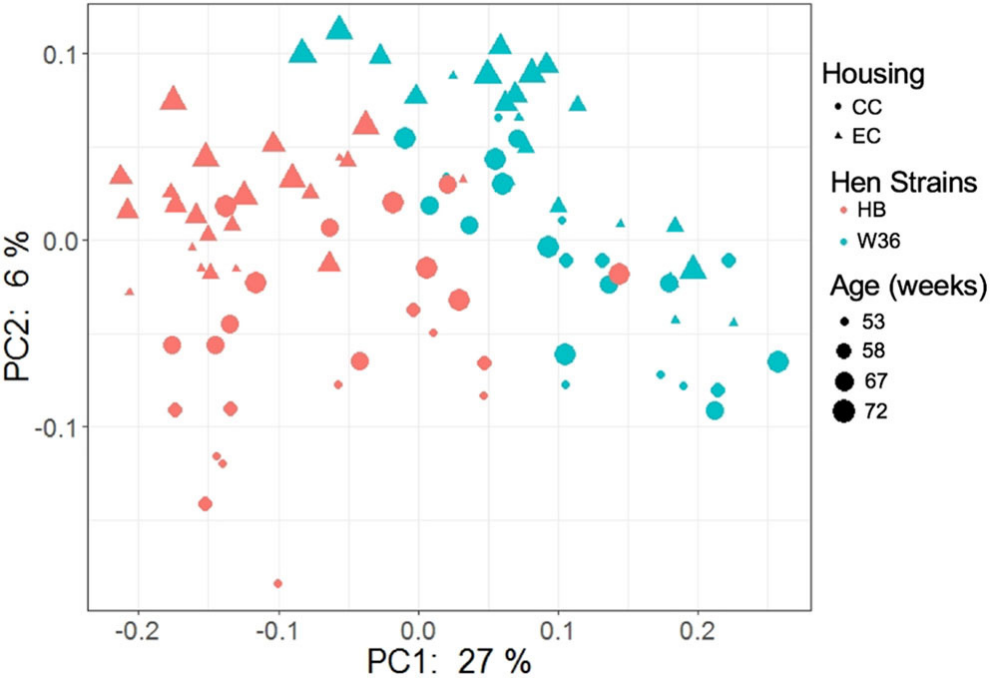
PCoA plot showing cecal microbiota community structure between two different housing (CC; Conventional Cage and EC; Enriched Colony Cage) and breed types (HB; Hy-Line Brown and W36; Hy-Line W-36) at 53, 58, 67, and 72 weeks of hens' ages. The plot was generated using unweighted distance metric.

**Figure 14:**
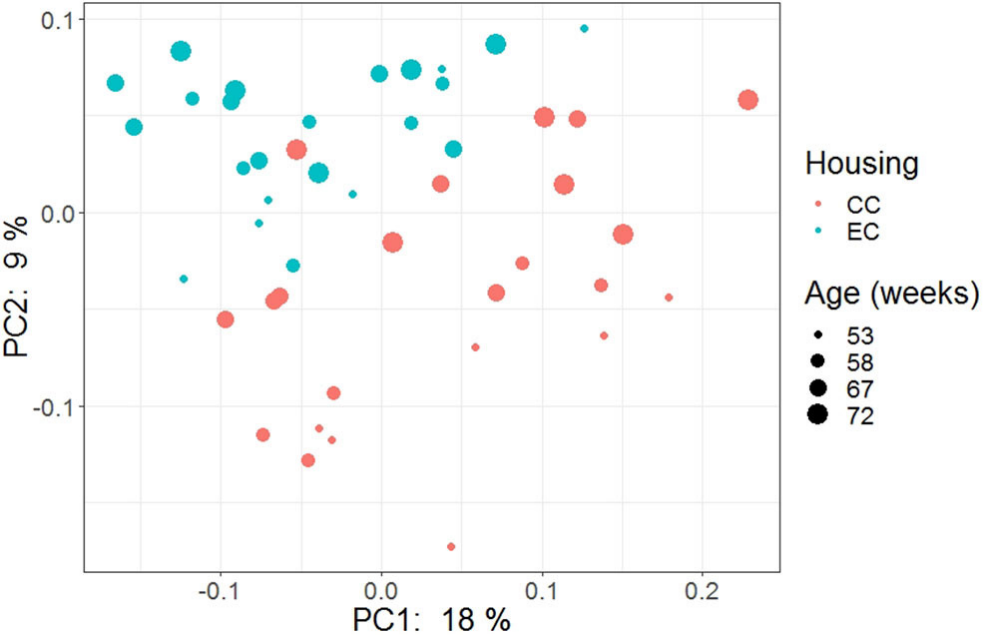
PCoA plot showing cecal microbiota community structure in Hy-Line Brown (HB) housed in Conventional Cage (CC) and Enriched Colony Cage (EC) systems.

**Figure 15:**
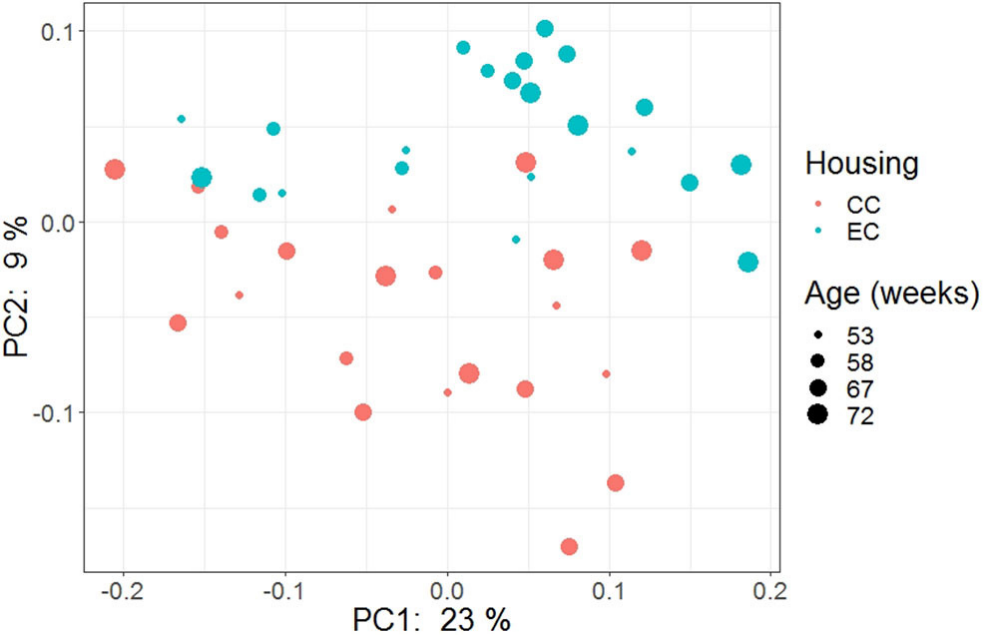
PCoA plot showing cecal microbiota community structure in Hy-Line W-36 (W36) housed in Conventional Cage (CC) and Enriched Colony Cage (EC) systems.

The beta diversity of two different strains and housing types across four-time points is shown in the PCoA plot (Figure 13). The PERMANOVA results showed that the microbial community structure in laying hens was significantly affected by all three variable analyzed; age (*P* = 0.028), housing (*P* = 0.001), and strain (*P* = 0.001). Pairwise PERMANOVA results showed that there was a tendency of microbial community structure difference between EC and CC throughout four ages, significant difference between EC and CC was observed only at 67 weeks of age. This is in accordance with the results in alpha diversity. Furthermore, in agreement with taxonomic composition and alpha diversity, the strain effect was most prominent on beta diversity among other variables since there was significant difference in beta diversity between HB and W36 throughout all four ages (adjusted *P* < 0.05). On the contrary to housing, increase in age resulted in significant difference in beta diversity even within the same strains, which was more noticeable in HB (53 vs. 67, 58 vs. 67 and 72, and 67 vs. 72) than W36 (53 vs. 67). Moreover, the cecal microbiota community structure was affected by housing types in both HB (Figure 14) and W36 hens (Figure 15) at *P* < 0.00.

### Functional Predictions of Cecal Microbiota

**Figure 16:**
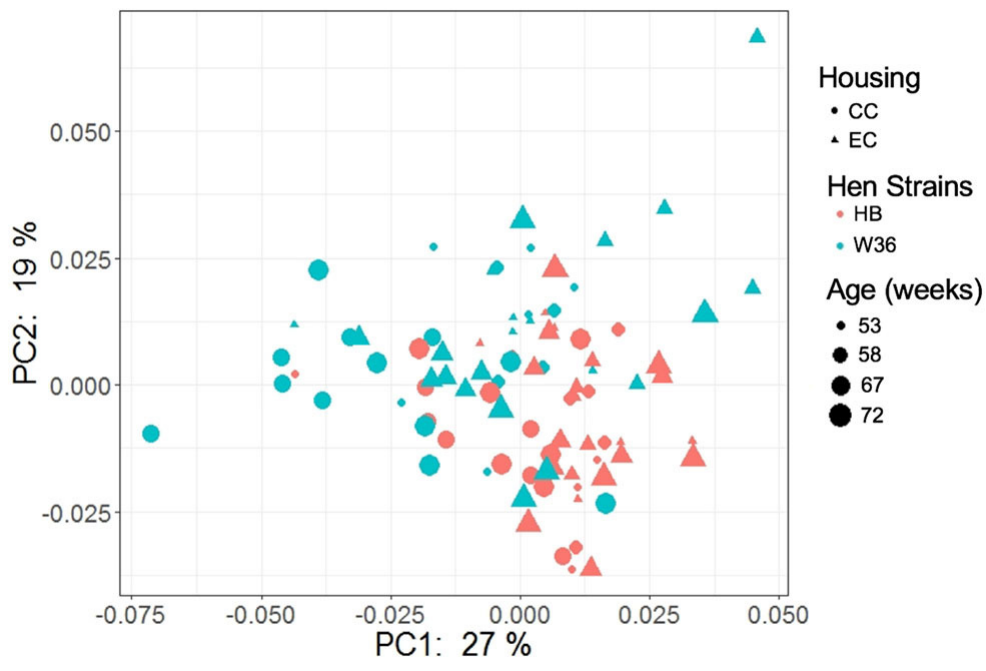
PCoA plot showing cecal microbiota functional diversity between two different housing (CC; Conventional Cage and EC; Enriched Colony Cage) and breed types (HB; Hy-Line Brown and W36; Hy-Line W-36) at 53, 58, 67, and 72 weeks of hens' ages. The plot was created using Bray Curtis distance metric generated from metabolic pathways predicted by PICRUSt2.

The PCoA plot illustrating the microbial functional diversity between two different housing and strain types across four different time intervals is shown in Figure 16. The factors such as age, housing, and strain not only affected community diversity but also affected the functional diversity of cecal microbiota (*P* < 0.001). However, functional diversity of cecal microbiota was less affected than their community structure by the strain as visualized in Figure 16, where the strain effect was significant at all ages except at 72 weeks (PERMANOVA pairwise, *P* < 0.05). On the contrary, housing types affected functional diversity more than the community structure, where there was significant difference in functional diversity between CC and EC at both 67 and 72 weeks (PERMANOVA pairwise, *P* < 0.05).

**Figure 17:**
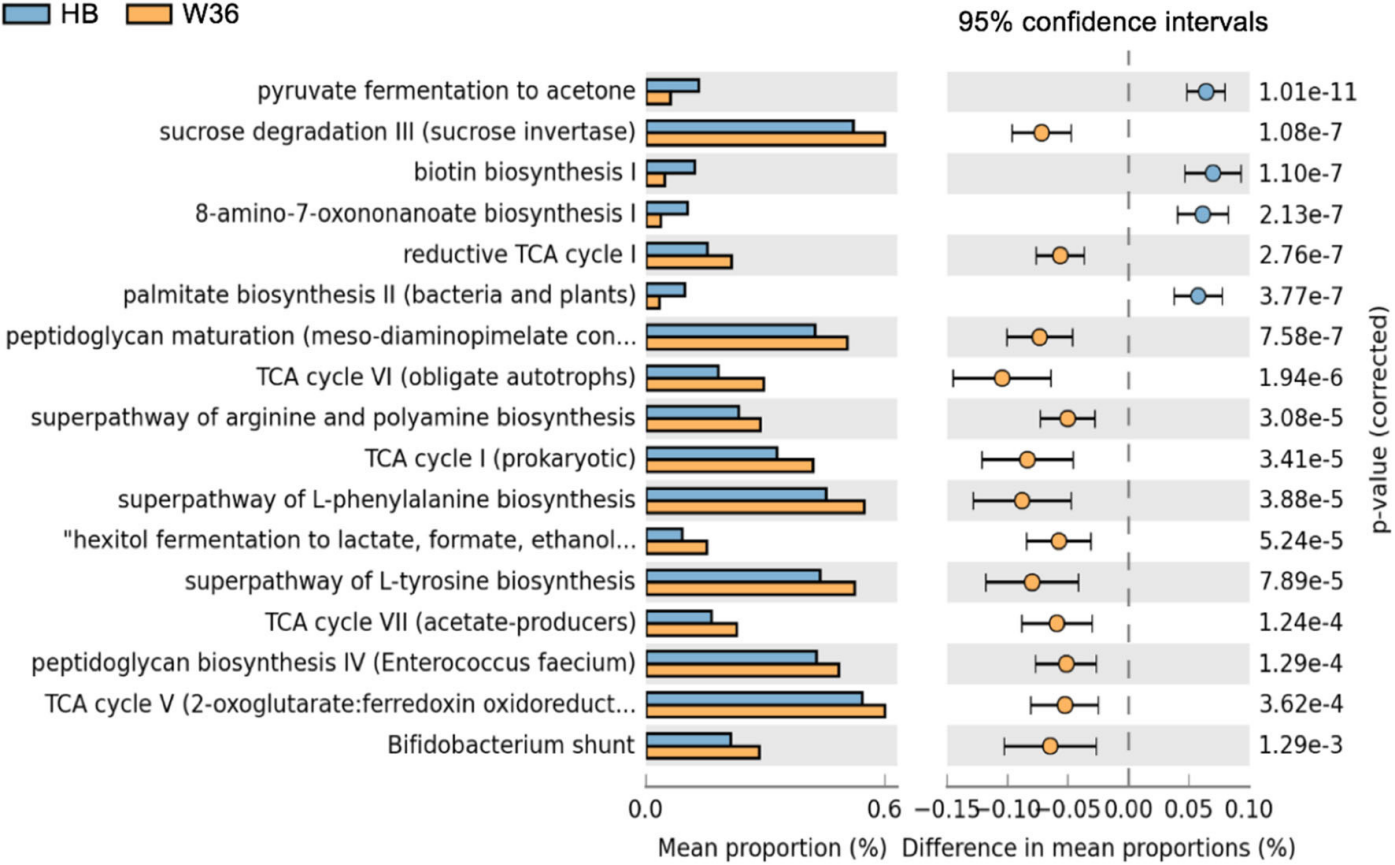
Differentially abundant metabolic pathways of cecal microbiota between Hy-Line Brown (HB) and Hy-Line W-36 (W36). STAMP software was used to identify differentially abundant features using Welch's *t*-test, where features were filtered using *P* > 0.05 and difference in mean proportions (%) <0.05 criteria.

Differentially abundant predicted metabolic pathways of cecal microbiota between HB and W36 hens are shown in Figure 17. Among 17 differentially abundant pathways between HB and W36, 13 pathways were significantly enriched in W36 while 4 pathways were significantly enriched in HB. In W36, metabolic pathways related to TCA cycle, sucrose degradation, hexitol fermentation (lactate, formate, and ethanol), amino acids biosynthesis (arginine, L-phenylalanine, and L-tyrosine), the *Bifidobacterium* shunt, and peptidoglycan biosynthesis were significantly enriched in W36. On the other hand, pathways related to pyruvate fermentation to acetone, and biotin synthesis, palmitate biosynthesis were highly abundant in HB (Figure 17).

**Figure 18:**
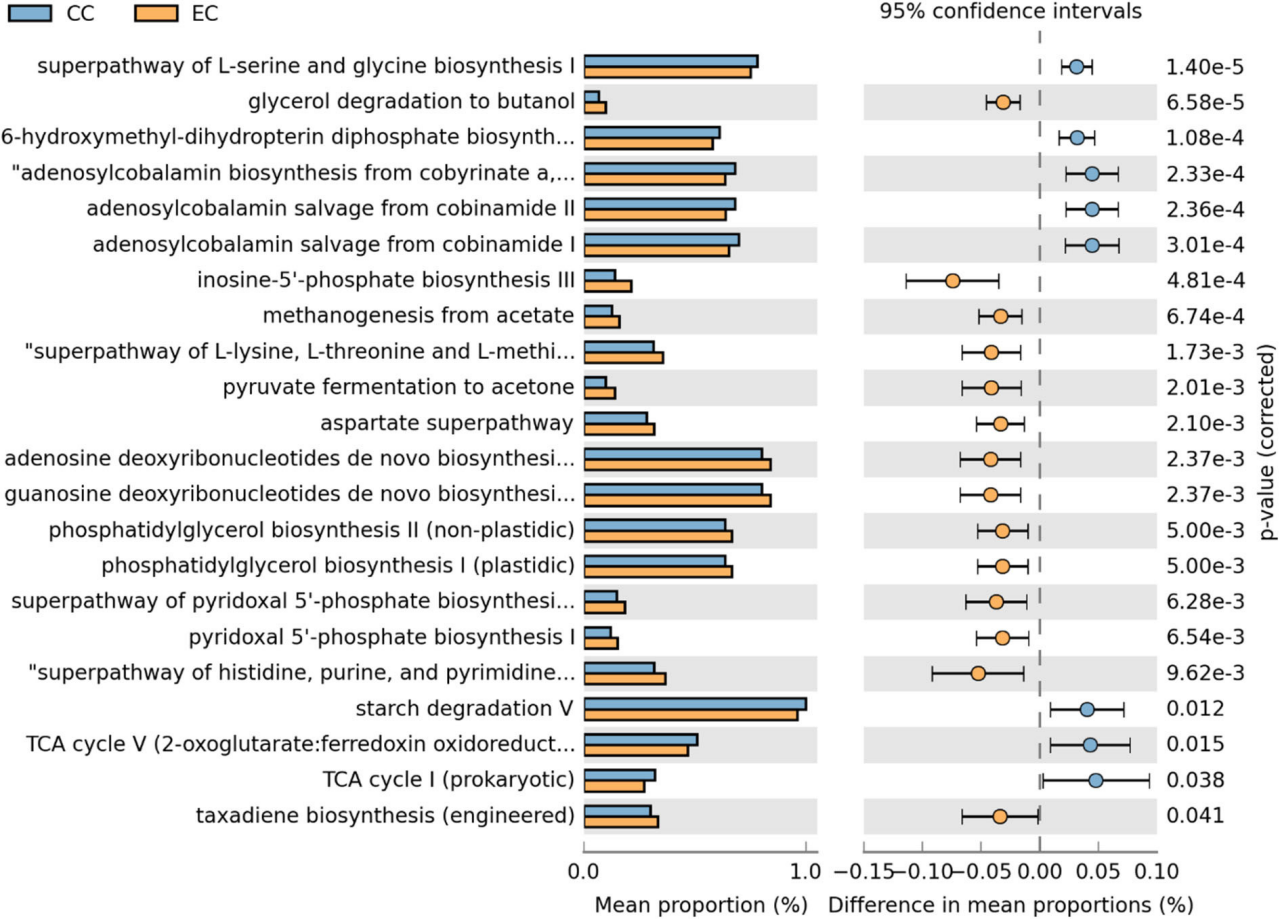
Differentially abundant metabolic pathways of cecal microbiota in Hy-Line Brown (HB) housed in Conventional Cage (CC) and Enriched Colony Cage (EC) systems. STAMP software was used to identify differentially abundant features using Welch's *t*-test, where features were filtered using *P* > 0.05 and difference in mean proportions (%) <0.03 criteria.

**Figure 19:**
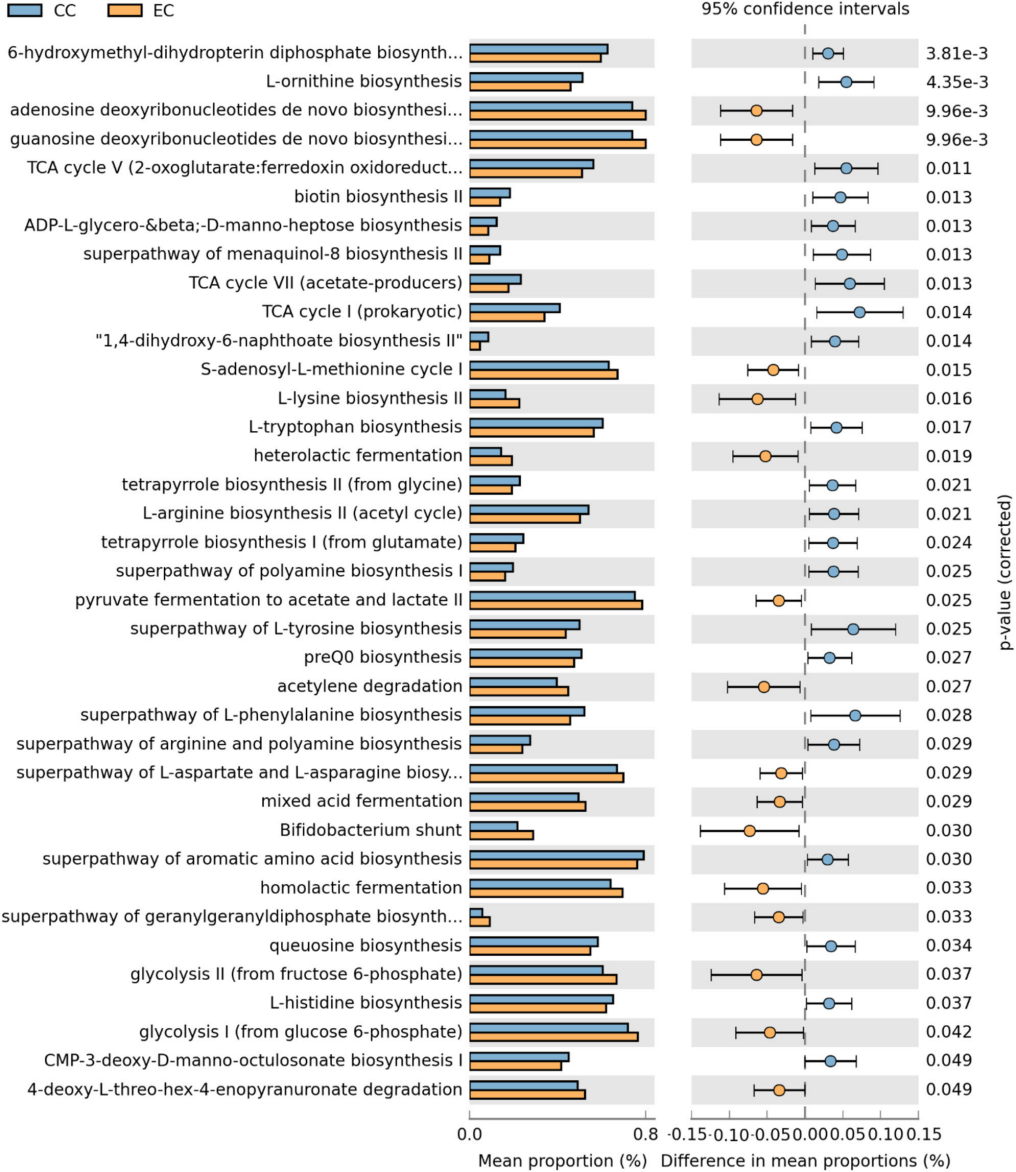
Differentially abundant metabolic pathways of cecal microbiota in Hy-Line W-36 (W36) housed in Conventional Cage (CC) and Enriched Colony Cage (EC) systems. STAMP software was used to identify differentially abundant features using Welch's *t*-test, where features were filtered using *P* > 0.05 and difference in mean proportions (%) <0.03 criteria.

Moreover, differentially abundant microbial metabolic pathways between CC and EC housing systems in HB and W36 laying hens are shown in Figures 18, 19, respectively. In HB group, altogether 22 metabolic pathways (8 in CC and 14 in EC) were differentially presented between CC and EC housing systems after filtering pathways with *P* > 0.05 (Welch's *t*-test) and effect size (% difference in mean proportions) <0.03 using STAMP (Figure 18). Specifically, pathways of TCA cycle, amino acid biosynthesis (L-serine and L-glycine), starch degradation, adenosylcobalamin (also known as vitamin B12 or coenzyme B12) biosynthesis, and 6-hydroxymethyl-dihydropterin diphosphate biosynthesis (precursor of vitamin B9 synthesis) were significantly enriched in CC group, whereas pathways of glycerol degradation, methanogenesis, amino acid biosynthesis (L-lysine, L-threonine, L-methionine, and L-aspartate), and purine and pyrimidine biosynthesis were significantly enriched in EC group.

In the W36 group, altogether 37 metabolic pathways (22 in CC and 15 in EC) were differentially presented between CC and EC housing systems as shown in Figure 19. Like in the HB group, pathways of TCA cycle and 6-hydroxymethyl-dihydropterin diphosphate biosynthesis (precursor of vitamin B9 synthesis) were significantly higher in CC group, while pathways of purine nucleotide and amino acids (L-lysine and L-aspartate) biosynthesis in EC group. On the contrary, biosynthesis pathways of amino acids such as L-ornithine, L-tryptophan, L-arginine, L-tyrosine, L-histidine, and L-phenylalanine were significantly enriched in CC group. Another important observation was significant enrichment of glycolysis, acid fermentation and *Bifidobacterium* shunt pathway in EC group, while significant enrichment of pathways associated with various vitamins biosyntheses such as K2 (menaquinol-8 biosynthesis) and B12 (tetrapyrrole biosynthesis I) was observed in CC group (Figure 19).

## Discussion

The intestinal microbiotas of chickens are affected by various factors such as age, breed, gut region, sex, feed, housing, hygiene, medication, temperature, litter, location, and maternal factors (13). Among these factors, the effect of feed on intestinal microbiota composition of chickens is widely studied. In laying hens, different dietary supplementations such as threonine (29), rapeseed meal (30), probiotics (31–33), calcium (34), and flaxseed oil (35) have been found to modulate the intestinal microbiota. However, there is very limited information regarding the changes in intestinal microbiota composition of laying hens due to the housing systems.

To our knowledge, this is the first study that reported the effects of CC and EC on alterations of cecal microbiota in two important commercial strains of laying hens, W36 and HB. In the present study, we found changes in cecal microbiota composition, their diversities and predicted functional pathways in both laying hen strains raised in CC and EC housing systems during the late production stage. A previous study reported a higher number of *Clostridium perfringens* in ileum and cecum of broiler chickens raised on organic farms as compared to the conventional farms was observed (36). However, they suggested that the lower count of *C. perfringens* in conventional farms might be achieved due to the application of Salinomycin in the conventional feed that has antibiotic properties. In addition, they found an increase in Lactobacilli, while a decrease in *Enterobacteriaceae* counts in the ileal contents of the chickens from organic farms (36). Another study reported enrichment of *Bifidobacterium* in both ileum and ceca of broiler chickens which were provided free daytime access to outdoor space as compared to those chickens which were kept at indoors range (37). Furthermore, both the composition and functions of cecal microbiota were different in Dagu chickens raised in a free-range setting as compared to those raised in cages (38). Firmicutes/Bacteroidetes ratio was higher in cecum of cage-raised chickens, while the abundance of Bacteroidetes was higher in free-range chickens (38). Although no direct comparisons can be made between the studies, we also reported the higher abundance of Bacteroidetes in EC where hens have more flexibility in movement and behavioral expression, while the higher abundance of Firmicutes in CC where they have restricted movement, especially at 67 weeks of age. In addition, we also reported significantly higher abundance of Proteobacteria in EC at 72 weeks of age in CC and EC. Many gram-negative pathogenic bacteria such as *Escherichia, Salmonella, Campylobacter, Helicobacter*, and *Vibrio* belong to the phylum Proteobacteria whose increase can be considered as a potential indicator of gut dysbiosis (39). This was also reflected at the genus level where *Campylobacter* and unknown genera of family Campylobacteraceae and Helicobacteraceae were significantly higher in EC at 72 weeks.

Xu et al. also reported a higher abundance of cecal microbiota functions associated with amino acids and glycan metabolic pathways in Dagu chickens from free-range (38). Recently, a study compared the cecal microbiota of You chickens (a Chinese native breed) reared in cages and free-range system at 45 weeks of age and reported the difference in their composition, diversity, and metabolic functions between the two systems (40). More specifically, the alpha diversity was decreased in chickens housed in cages as compared to those from free-range. In addition, most of the KEGG pathways of cecal microbiota associated with various functions such as metabolism, alkaloid biosynthesis, and amino acids degradation were down-regulated in cages-reared chickens. In this study, the alpha diversity was significantly higher in EC as compared to CC at 67 weeks of age and was numerically higher throughout all ages. Likewise, several metabolic pathways were differentially enriched between CC and EC in the current study, which were further dependent on laying hen strains. For instance, 22 metabolic pathways (8 in CC and 14 in EC) were differentially abundant in HB strain, while 37 metabolic pathways (22 in CC and 15 in EC) in W36 strain, suggesting more pronounced effects of housing in W36. Specifically, pathways related to energy and nucleotide metabolism, and amino acids and vitamin B biosynthesis were differentially presented between two housing systems in a strain-dependent manner.

The phylum Actinobacteria and its genus *Bifidobacterium* were significantly enriched in W36 as compared to the HB throughout all four-time points. Bifidobacteria are common probiotic bacteria whose effects on hosts' health and diseases are studied elsewhere (41, 42), and are widely considered to confer beneficial effects on hosts through their metabolic activities. Specifically, bifidobacteria are well-known for their ability to ferment complex carbohydrates in the lower part of the intestine that bypasses the degradation in the upper parts through various carbohydrate-degrading enzymes (43). They can ferment diverse complex carbon sources including gastric mucin, (trans)-galactooligosaccharides, xylo-oligosaccharides, malto-oligosaccharides, fructo-oligosaccharides, pectin, soybean oligosaccharides, and other plant derived-oligosaccharides. However, their ability to degrade particular carbon source is species/strain-dependent (44). Through fermentation, bifidobacteria can degrade complex carbohydrates to monosaccharides which are further degraded to intermediates of the hexose fermentation pathway (also known as Bifidobacterium shunt or fructose-6-phosphate shunt) (45) and finally converted to short-chain fatty acids, especially acetate and lactate (42). In the current study, carbohydrate degradation was significantly enriched in W36 as compared to the HB. In addition, the *Bifidobacterium* shunt pathway was significantly enriched in W36 as compared to the HB. The anti-Campylobacter activity of *Bifidobacterium* was also previously reported in poultry (46).

Similarly, butyrate-producing genera such as *Butyricicoccus* and *Subdoligranulum* were significantly higher in W36 as compared to HB at 58 and 72 weeks. Butyrate, a metabolite of intestinal microbiota is considered as an important feed additive in animal production due to its several beneficial effects such as improvement of performance parameters and maintenance of gut health by controlling the proliferation of bacterial pathogens and enhancement of intestinal development (47, 48). Other important observations were time-dependent enrichment of phyla Synergistetes and Spirochaetes and genera such as *Clostridium* and *Paraprevotella* in HB as compared to W36. Briefly, there were 36, 21, 54, and 7 differentially abundant genera between HB and W36 at 53, 58, 67, and 72 weeks, respectively. Interestingly, the differences in cecal microbiota between W36 and HB were not only observed in their composition but also in both community and functional diversities. Significant interaction effect of housing and laying hen strains on egg production were also reported earlier (49).

In sum, cecal microbiota composition, diversities, and their functional pathways were affected by housing type which further varied between two commercial laying hen strains, HB and W36. This suggests that both housing and strains should be considered for selection of the two major laying hen housing systems.

## Data Availability Statement

Sequencing data of cecal microbiota is available on NCBI Sequence Read Archive under BioProject number PRJNA627663.

## Ethics Statement

The animal study was reviewed and approved by Institutional Animal Care and Use Committee (IACUC) at Mississippi State University (AUP 17-554).

## Author Contributions

PA planned, designed and executed the animal experiment, and contributed to editing the final version of the manuscript. BA analyzed data and wrote the first version of the manuscript. PA, AK, BA, S-RJ, and YK revised the manuscript. All authors reviewed and finally approved the manuscript.

## Conflict of Interest

The authors declare that the research was conducted in the absence of any commercial or financial relationships that could be construed as a potential conflict of interest.
